# Successful treatment of localized scleroderma using brodalumab: A case report

**DOI:** 10.1177/2050313X251341561

**Published:** 2025-05-14

**Authors:** Alexandra Carla Bobica, Catherine Zhu, Catherine Silotch, Hessah BinJadeed, Elena Netchiporouk

**Affiliations:** 1 Division of Clinical and Translational Research, McGill University, Montreal, QC, Canada; 2 Faculty of Medicine, McGill University, Montreal, QC, Canada; 3 Division of Dermatology, McGill University Health Centre, Montreal, QC, Canada

**Keywords:** localized scleroderma, morphea, cancer, immune checkpoint inhibitors, brodalumab

## Abstract

We report the first documented use of brodalumab, an IL-17 receptor antagonist, for treating severe linear localized scleroderma refractory to conventional therapies, including methotrexate, mycophenolate mofetil and corticosteroids. The patient demonstrated rapid and sustained improvement in disease activity and severity, highlighting the potential role of IL-17 signaling in the pathogenesis of localized scleroderma. This case supports further investigation of IL-17 pathway blockade in autoimmune fibrotic diseases.

## Introduction

Localized scleroderma (LS), also known as morphea, is an autoimmune fibrotic skin disease.^1^ It is classified into circumscribed, linear, generalized, pansclerotic and mixed forms.^1^ While circumscribed LS usually follows a benign and self-resolving course, other forms are associated with a risk of permanent disfigurement and/or disablement.^1^ There are currently no guidelines for the management of LS in adult patients.^1^ Based on expert opinion, active LS with the potential for disability is treated with systemic immunosuppressive agents such as methotrexate or mycophenolate mofetil, alone or in combination with systemic corticosteroids.^2^ However, real-world efficacy ranges from 50% to 70% and these therapies are accompanied by adverse events.^2^ We report a challenging case of severe active linear LS in a cancer patient who has failed conventional systemic agents in combination with prednisone but responded rapidly to brodalumab therapy (an IL-17 receptor antagonist).

## Case report

A female patient in her 40s presented to our clinic in November 2023 for the management of extensive linear LS in the context of active malignancy. Her medical history included primary breast and lung cancers and cardiac ablation for arrhythmia. LS, initially circumscribed subtype, was first diagnosed in 2005 and remitted with ultrapotent topical steroids. In 2019, following the initiation of immunotherapy and chemotherapy for lung cancer, LS reactivated and progressed to a linear subtype. Sequential treatments with methotrexate and mycophenolate mofetil failed to control disease activity and progression. While immunotherapy and chemotherapy were discontinued in 2020 based on favorable cancer evolution, LS progression persisted. At presentation, LS plaques extended from the left shoulder down to the arm, the abdomen and the left lower extremity down to the toes with associated dermal and muscle atrophies. To control disease activity and halt progression, systemic steroids were initiated (1 mg/kg tapered over 6 weeks) and topical roflumilast 0.3% cream was added as an adjuvant. She was referred to hematology–oncology for consideration of extracorporeal photopheresis (ECP). In the interim, she continued weekly physical therapy. Upon reassessment in December 2023, systemic steroids resulted in the reduction of disease activity and progression, however, coverage for ECP was declined. The treatment plan was modified and consisted of tapering off prednisone while maintaining the use of topicals and physiotherapy. Upon re-examination in February 2024, increased erythema, pain, itch and new expanded plaques were noted (physician global assessment-activity (PGA-A): 30, PGA-damage (PGA-D): 60; Figure 1(a)). Off-label compassionate brodalumab therapy (210 mg every 2 weeks) was approved and initiated in March 2024. At follow-up in May 2024, substantial improvement in disease activity and severity was noted (PGA-A: 5, PGA-D: 40; Figure 1(b)), with complete relief of pain. The patient discontinued all other therapies. At last follow-up in January 2025, LS remained well controlled on brodalumab monotherapy (PGA-A: 5, PGA-D: 40).

**Figure 1.**
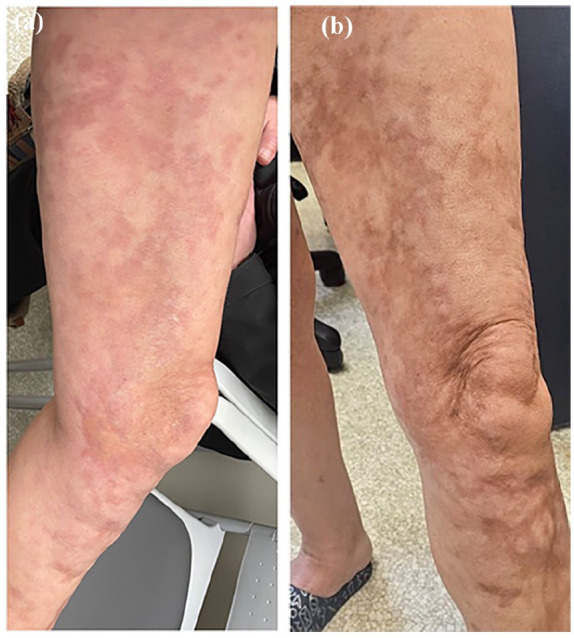
Clinical evolution of linear morphea before and after brodalumab treatment. (a) Prior to brodalumab treatment, violaceous patches and atrophic plaques are observed following a linear distribution. An expanding edematous edge is noted medial to the left knee, indicative of active disease progression. (b) Two months after initiating brodalumab therapy, erythema is resolved and replaced by post-inflammatory hyperpigmentation and atrophic damage, with no evidence of active inflammation.

## Discussion

Severe LS, particularly when induced by immunotherapy in oncology patients, presents a significant treatment challenge. Prednisone doses exceeding 20 mg are known to hamper the effectiveness of immunotherapy, while the safety of alternative off-label therapies, such as tocilizumab and JAK inhibitors, in cancer patients is debated. This case report exemplifies brodalumab’s promising effectiveness and safety in difficult-to-treat LS. The importance of IL-17 in fibrotic diseases has previously been suggested.^3^ Elevated levels of IL-17A and increased Th17 cell populations have been observed in early systemic sclerosis (SSc) patients, suggesting a role in the pathogenesis of immune-mediated fibrotic diseases.^3^ IL-17A was shown to promote fibrosis by stimulating fibroblast proliferation and collagen production.^3^ In addition, IL-17A can synergize with other cytokines, such as transforming growth factor-beta, to enhance fibrotic response.^3^ Although less extensively studied, elevated levels of IL-17A have been observed in LS patients.^4^

While this is an initial report of brodalumab use in LS, a recent phase 3 placebo-controlled, double-blinded trial of 100 SSc patients demonstrated a rapid and sustained improvement in skin fibrosis.^5^ Specifically, there was a 21.2 point reduction in the modified Rodnan skin score at week 24 from baseline (95% confidence interval, −23.9 to −18.5; *p* < 0.0001).^5^ In addition, brodalumab treatment suppressed the development of digital ulcers, the deterioration of respiratory function and the progression of lung fibrosis.^5^ It also improved the symptoms of gastroesophageal reflux disease and chronic illness therapy-fatigue score, with no significant safety concerns.^5^

This case report highlights the potential role of IL-17 pathway blockade in the treatment of immune-mediated fibrotic diseases such as LS, particularly when other options fail or are unsuitable. Future research on the role of IL-17 signaling in LS pathogenesis and clinical trial data investigating the efficacy of brodalumab in LS are needed.
